# Association of Systemic Inflammatory Indices With Breast Cancer: A Cross-Sectional Analysis of National Health and Nutrition Examination Survey (NHANES) Data

**DOI:** 10.7759/cureus.110507

**Published:** 2026-06-09

**Authors:** Aqsa Kiran, Haseeb Nasir, Sarmad Shaikh, Elsa Zainab Ansari, Aimen Luqman, Ayesha Urooj

**Affiliations:** 1 Public Health, Anglia Ruskin University, Chelmsford, GBR; 2 Internal Medicine, People's University of Medical and Health Sciences for Women, Nawabshah, PAK; 3 Internal Medicine, Southampton General Hospital NHS Foundation Trust, Southampton, GBR; 4 Internal Medicine, Military Hospital, Rawalpindi, PAK; 5 General Surgery, Jinnah Postgraduate Medical Centre (JPMC), Karachi, PAK; 6 Diagnostic Radiology, Combined Military Hospital, Multan, PAK; 7 Internal Medicine, People's Primary Healthcare Initiative (PPHI) Sindh, Thatta, PAK

**Keywords:** breast neoplasms, inflammations, leukocyte, neutrophil, platelet counts

## Abstract

Background: Breast cancer remains one of the leading causes of cancer-related morbidity and mortality among women worldwide. Systemic inflammatory indices derived from routine hematological parameters, including NLR, PLR, and SII, have recently gained attention as potential biomarkers associated with breast cancer risk and progression. However, population-based evidence evaluating these inflammatory markers after adjustment for demographic, socioeconomic, and lifestyle-related confounders remains limited.

Aim: We evaluated the association of systemic inflammatory indices, including NLR, PLR, and SII, with self-reported history of breast cancer in a nationally representative cross-sectional population sample after adjustment for demographic, socioeconomic, and lifestyle-related factors.

Methods: This cross-sectional analytical study utilized data from the NHANES 2021-2023 cycle. Female participants aged ≥18 years with available complete blood count parameters and breast cancer status information were included. Breast cancer status was defined using self-reported physician diagnosis. NLR, PLR, and SII were calculated from hematological parameters obtained through standardized laboratory assessment. Demographic, socioeconomic, and lifestyle variables included age, body mass index (BMI), race/ethnicity, poverty-income ratio (PIR), smoking history, and alcohol intake. Due to non-normal distribution, continuous variables were summarized as median (interquartile range) and compared using Mann-Whitney U tests. Categorical variables were analyzed using Rao-Scott adjusted chi-square tests accounting for the NHANES complex survey design. Multivariable survey-weighted logistic regression models were constructed separately for NLR, PLR, and SII, adjusting for potential confounders. Adjusted odds ratios (aORs) with 95% confidence intervals (CIs) were reported, and p < 0.05 was considered statistically significant.

Results: A total of 5,298 women were included, of whom 150 (weighted prevalence ~1.9%) reported a history of breast cancer. Women with breast cancer were older than those without breast cancer. Exploratory unweighted descriptive comparisons showed higher median NLR, PLR, and SII values among participants with breast cancer compared with controls. However, after adjustment for demographic, socioeconomic, and lifestyle factors in survey-weighted multivariable models, none of the inflammatory indices demonstrated an independent association with breast cancer. Age consistently remained the strongest independent predictor of breast cancer across all models (p < 0.001).

Conclusion: Although exploratory unweighted analyses demonstrated higher inflammatory index values among women with breast cancer, these associations were not independently significant in survey-weighted multivariable models.

## Introduction

Breast cancer is one of the leading causes of mortality due to cancer among women worldwide [1,2]. Among all the female cancers, it is present in around 25% of the cases [3]. In 2022, it was reported that approximately 2.3 million new cases and 670,000 deaths were due to breast cancer worldwide [4,5]. The incidence rate of breast cancer is increasing across the globe, with around a 1-5% rise annually [6]. As per the future projections, it is expected that there will be a 38% increase in new cases and a 68% rise in mortality by 2050 [7].

As breast cancer cases continue to rise, the need for reliable, accessible, and cost-effective investigations has also increased [8]. Such investigations may aid in early detection and risk stratification [9]. However, conventional serum markers such as CA15-3 and carcinoembryonic antigen have limited sensitivity and specificity, due to which they have limited use in early detection of breast cancer [10]. Ongoing studies suggest that systemic inflammation also plays a role in breast cancer development and progression [11,12]. Hematological indices, which reflect the immune-inflammatory cascade, therefore have the potential for diagnostic and prognostic indication in breast cancer [13].

Several systemic inflammatory markers are derived from routine complete blood counts, including the neutrophil-to-lymphocyte ratio (NLR), platelet-to-lymphocyte ratio (PLR), and systemic immune-inflammation index (SII) [14,15]. Literature suggests that elevated NLR and PLR are associated with poor prognosis and reduced survival across multiple malignancies [16]. Similarly, SII is also considered a composite marker that reflects systemic inflammatory burden [17]. Breast cancer risk is also influenced by various demographic, socioeconomic, and lifestyle factors [18,19]. Age, body mass index (BMI), race/ethnicity, income-to-poverty ratio, smoking status, and alcohol consumption have all been independently associated with breast cancer risk [20]. However, there is very limited evidence that has evaluated the relationship between these inflammatory indices and breast cancer in the context of these confounders. As these indices are derived from routinely available complete blood count parameters, they may represent accessible and cost-effective markers for population-level risk stratification and identification of individuals who may benefit from further evaluation.

The National Health and Nutrition Examination Survey (NHANES) data provide an opportunity to analyze these associations in a large, nationally representative sample of the population. Therefore, the present study aimed to evaluate the association of systemic inflammatory markers (NLR, PLR, and SII) with breast cancer status while adjusting for key demographic, socioeconomic, and lifestyle factors using NHANES 2021-2023 data. This study was designed as an exploratory association analysis and not as a predictive biomarker validation study. We hypothesized that higher inflammatory index values would be independently associated with a history of breast cancer after adjustment for potential confounding factors.

## Materials and methods

Study design and data source

This cross-sectional analytical study was performed using publicly available data from the August 2021 to August 2023 cycle of the National Health and Nutrition Examination Survey (NHANES) [21]. The 2021-2023 NHANES cycle was selected because it is the most recent nationally representative dataset that includes both complete blood count measurements required to calculate inflammatory indices and questionnaire data on breast cancer history. NHANES is a population-based survey conducted by the National Center for Health Statistics (NCHS), Centers for Disease Control and Prevention (CDC). Data collection in NHANES consists of standardized home interviews, laboratory investigations, and physical examinations conducted in specially equipped mobile centers. For the present analysis, demographic, laboratory, examination, smoking, alcohol, and medical conditions datasets were merged using the unique participant identification number (SEQN).

Study population

Female participants aged 18 years and older with available complete blood count parameters and breast cancer status information were considered eligible for inclusion. Individuals with incomplete data for primary study variables, including neutrophil count, lymphocyte count, platelet count, age, or body mass index (BMI), were excluded from relevant statistical analyses. Participants with hematological values insufficient for the calculation of inflammatory indices were similarly excluded. The final analytic cohort consisted of eligible participants with complete demographic, laboratory, and questionnaire-based information required for the study objectives. A complete case analysis approach was employed. Participants with missing data for variables required in a given analysis were excluded from that analysis. No imputation procedures were performed.

Study variables

Breast Cancer Status

The primary outcome variable was self-reported physician-diagnosed history of breast cancer (hereafter referred to as breast cancer status). It was obtained from the NHANES medical conditions questionnaire variable MCQ230A (Ever told you had cancer or malignancy), together with the cancer type variable identifying breast cancer among respondents reporting a cancer diagnosis. Individuals reporting a history of breast cancer were categorized into the breast cancer group, whereas participants without any reported breast cancer diagnosis were categorized into the control group.

Inflammatory Markers

The primary inflammatory markers evaluated in the study included the neutrophil-to-lymphocyte ratio (NLR), platelet-to-lymphocyte ratio (PLR), and systemic immune-inflammation index (SII). Total white blood cell count, neutrophil percentage, lymphocyte percentage, and platelet count were obtained from the NHANES Complete Blood Count laboratory dataset (variable codes LBXWBCSI, LBXNEPCT, LBXLYPCT, and LBXPLTSI, respectively). All of the markers were derived from complete blood count laboratory measurements. Absolute neutrophil and lymphocyte counts were estimated using total white blood cell count and differential leukocyte percentages available within the NHANES laboratory dataset. NLR was calculated by dividing the absolute neutrophil count by the absolute lymphocyte count. PLR was calculated by dividing the platelet count by the absolute lymphocyte count. SII was calculated using the formula:

SII = Platelet Count × Neutrophil Count divided by Lymphocyte Count

Demographic and Lifestyle Variables

**Figure 1:**
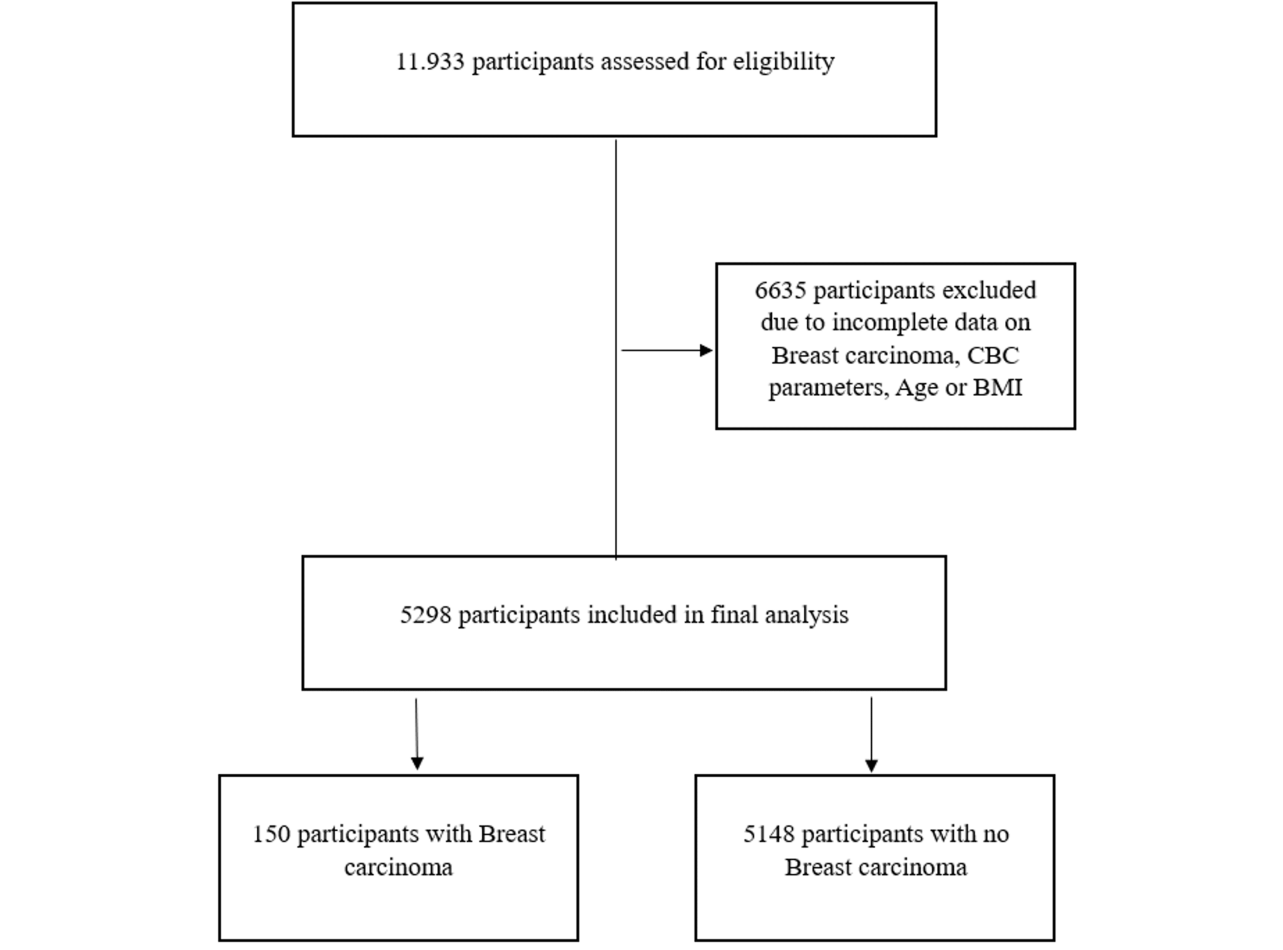
Flowchart of study selection

Age was recorded in years at the time of survey participation. BMI was calculated by NHANES using measured height and weight and expressed in kilograms per meter squared (kg/m²). Race/ethnicity was categorized according to NHANES-defined classifications, including non-Hispanic White, non-Hispanic Black, Hispanic, non-Hispanic Asian, and other or multiracial groups. Socioeconomic status was assessed using the family income-to-poverty ratio (PIR), a standardized measure comparing household income with the federal poverty threshold after adjustment for household size and survey year. Smoking status was determined using questionnaire data regarding lifetime cigarette exposure. Participants reporting smoking at least 100 cigarettes during their lifetime were categorized as smokers, whereas those reporting fewer than 100 lifetime cigarettes were categorized as non-smokers. Alcohol intake was assessed using NHANES questionnaire data regarding heavy alcohol consumption patterns. Female participants who reported consuming four or more drinks per day on a regular basis were categorized as having heavy alcohol intake, whereas all other participants were categorized as non-heavy alcohol consumers. Figure 1 shows patient selection.

Statistical analysis

Statistical analyses were performed using IBM Corp. Released 2019. IBM SPSS Statistics for Windows, Version 25. Armonk, NY: IBM Corp. All analyses accounted for the complex multistage probability sampling design of NHANES using the Complex Samples module. As the present study utilized laboratory and examination-based variables, Mobile Examination Center (MEC) weights (WTMEC2YR) were applied in accordance with NHANES analytic guidelines to ensure nationally representative estimates. Stratification (SDMVSTRA) and primary sampling units (SDMVPSU) were incorporated into all analyses. Continuous variables were assessed for normality using the Shapiro-Wilk test and summarized as median and interquartile range (IQR) due to non-normal distribution. As NHANES does not provide survey-weighted nonparametric procedures for median comparisons, unweighted Mann-Whitney U tests were used only for exploratory comparisons and not inferential population estimates. Categorical variables were analyzed using Rao-Scott adjusted chi-square tests. Multivariable survey-weighted logistic regression analyses were performed using the Complex Samples module to evaluate independent associations between inflammatory markers and breast cancer status. Separate regression models were constructed for NLR, PLR, and SII to minimize multicollinearity. Models were adjusted for age, BMI, race/ethnicity, poverty-income ratio, smoking history, and alcohol intake. Results were reported as adjusted odds ratios (aORs) with 95% confidence intervals, and a two-tailed p-value <0.05 was considered statistically significant.

Ethical considerations

The study utilized publicly accessible NHANES data. Statistical analysis was done using this de-identified NHANES data and therefore did not require additional institutional ethical approval. This study was reported in accordance with the Strengthening the Reporting of Observational Studies in Epidemiology (STROBE) guidelines for cross-sectional studies.

## Results

A total of 5,298 female participants from the NHANES 2021-2023 dataset were included in the final analysis. Among the included participants, 150 women reported a history of breast cancer, corresponding to an estimated weighted population prevalence of approximately 1.9%, whereas 5,148 participants had no history of breast cancer. Women with breast cancer were older compared to those without breast cancer. Whereas BMI did not differ much between breast cancer and non-breast cancer participants, as shown in Table 1.

**Table 1:** Comparison of baseline clinical variables between breast cancer and non-breast cancer participants (n=5298) An unweighted Mann-Whitney U test was applied for exploratory descriptive comparisons only.

Variable	Breast Cancer (n=150)	No Breast Cancer (n=5148)	Statistic value	p-value
Age (years) – Median (IQR)	70.00 (65.00 – 77.00)	53.00 (37.00 - 65.00)	147128.00	<0.001
BMI (kg/m²) - Median (IQR)	28.90 (24.30 – 34.60)	28.50 (24.70 – 33.70)	359974.50	0.270
Poverty-income ratio - Median (IQR)	3.30 (1.71-5.00)	2.80 (1.40 – 5.00)	262505.50	0.020

Race/ethnicity distribution differed significantly between participants with and without breast cancer based on Rao-Scott adjusted chi-square testing (p=0.001). The majority of weighted breast cancer cases were observed among non-Hispanic White women. Smoking history demonstrated a statistically significant association with breast cancer status after accounting for the complex survey design. Alcohol intake was not significantly associated with breast cancer status (p=0.742), as shown in Table 2.

**Table 2:** Comparison of categorical demographic and clinical variables between breast cancer and non-breast cancer participants (n=5298) Rao-Scott adjusted chi-square testing is applied

Variable	No Breast Cancer (n=5148)	Breast Cancer (n=150)	Statistics value	p-value
Race/Ethnicity				
Mexican American, n (%)	712 (13.7)	14 (8.1)	14.927	0.001
Other Hispanic, n (%)	569 (10.3)	10 (4.2)		
Non-Hispanic White, n (%)	2865 (57.8)	107 (76.7)		
Non-Hispanic Black, n (%)	689 (11.7)	12 (7.5)		
Non-Hispanic Asian/Others, n (%)	313 (6.6)	7 (3.5)		
Smoking history				
Yes, n (%)	3087 (63.5)	73 (43.7)	16.829	0.025
No, n (%)	2055 (36.4)	77 (56.3)		
Refused, n (%)	2 (0.03)	-		
Don’t Know, n (%)	4 (0.1)	-		
Alcohol intake				
Yes, n (%)	3235 (81.7)	106 (86.5)	1.226	0.742
No, n (%)	771 (18.2)	17 (13.5)		
Refused, n (%)	1 (0.001)			
Don’t Know, n (%)	5 (0.1)			

In exploratory unweighted comparisons using the Mann-Whitney U test, participants with breast cancer demonstrated higher median NLR, PLR, and SII values compared to participants without breast cancer. These analyses were descriptive and not intended as survey-weighted population inferences, as shown in Table 3.

**Table 3:** Comparison of NLR, PLR, and SII between breast cancer and non-breast cancer participants (n=5298) * Unweighted Mann-Whitney U test applied for exploratory descriptive comparisons only; results are not survey-weighted population estimates. NLR: neutrophil-to-lymphocyte ratio, PLR: platelet-to-lymphocyte ratio, SII: systemic immune-inflammation index

Variable	Breast Cancer (n=150)	No Breast Cancer (n=5148)	Statistic value	p-value
NLR – Median (IQR)	2.34 (1.57 – 2.96)	1.96 (1.49 – 2.63)	285063.50	0.001
PLR - Median (IQR)	140.23 (104.79 – 183.21)	128.85 (102.78 – 160.71)	293977.50	0.008
SII - Median (IQR)	566.05 (369.74 – 796.54)	491.59 (354.76 – 694.06)	293870.00	0.008

Following adjustment for age, race/ethnicity, BMI, poverty-income ratio, alcohol intake, and smoking history in multivariable logistic regression analysis, NLR did not demonstrate an independent association with breast cancer status (Exp[B]: 0.886, 95% CI: 0.698-1.124, p=0.295). Increasing age remained significantly associated with breast cancer in the adjusted model (Exp[B]: 1.109, 95% CI: 1.091-1.127, p < 0.001). No significant independent associations were observed for race/ethnicity categories, BMI, poverty-income ratio, alcohol intake, or smoking history, as shown in Table 4.

**Table 4:** Multivariable logistic regression analysis for association between NLR and breast cancer status (n=5298) *Weighted regression analysis done NLR: neutrophil-to-lymphocyte ratio, BMI: Body mass index

Variable	Adjusted OR	95% CI	p-value
NLR	0.886	0.698–1.124	0.295
Age	1.109	1.091–1.127	<0.001
Race/Ethnicity			
Non-Hispanic White	1.634	0.312-5.703	0.566
Other Hispanic	1.085	0.200-5.428	0.940
Mexican American	1.203	0.212-5.113	0.859
Non-Hispanic Black	0.861	0.145-3.966	0.861
BMI	1.026	0.981–1.073	0.237
Alcohol intake	1.727	0.905–3.297	0.092
Smoking history	0.580	0.315–1.068	0.077
Poverty-income ratio	1.036	0.918–1.170	0.544

Similarly, no statistically significant independent association was observed for PLR after adjustment for demographic, socioeconomic, and lifestyle-related covariates. Increasing age remained the only consistently significant predictor across the adjusted regression model, as shown in Table 5.

**Table 5:** Multivariable logistic regression analysis for association between PLR and breast cancer status (n=5298) *Weighted regression analysis done PLR: platelet-to-lymphocyte ratio, BMI: Body mass index

Variable	Adjusted OR	95% CI	p-value
PLR	1.000	0.996–1.004	0.904
Age	1.107	1.089–1.125	<0.001
Race/Ethnicity			
Non-Hispanic White	1.572	0.275-8.992	0.589
Other Hispanic	1.083	0.114-10.328	0.941
Mexican American	1.150	0.132-10.028	0.893
Non-Hispanic Black	0.887	0.150-5.257	0.887
BMI	1.025	0.980–1.073	0.258
Alcohol intake	1.759	0.920–3.362	0.083
Smoking history	0.585	0.322–1.063	0.075
Poverty-income ratio	1.032	0.915–1.164	0.583

After transforming SII into 100-unit increments and adjusting for demographic, socioeconomic, and lifestyle-related covariates, no statistically significant independent association was observed between SII and breast cancer status (aOR: 0.978, 95% CI: 0.923-1.038, p=0.442). Increasing age again remained significantly associated with breast cancer in the final adjusted model (p<0.001), as shown in Table 6.

**Table 6:** Multivariable logistic regression analysis for association between SII/100 and breast cancer status (n=5298) *Weighted regression analysis done SII: systemic immune-inflammation index, BMI: Body mass index

Variable	Adjusted OR	95% CI	p-value
SII/100	0.978	0.923–1.038	0.442
Age	1.108	1.090–1.126	<0.001
Race/Ethnicity			
Non-Hispanic White	1.598	0.269-9.479	0.583
Other Hispanic	1.078	0.111-10.472	0.945
Mexican American	1.178	0.130-10.696	0.876
Non-Hispanic Black	0.871	0.147-5.183	0.872
BMI	1.026	0.981–1.073	0.248
Alcohol intake	1.753	0.915–3.358	0.086
Smoking history	0.581	0.315–1.068	0.077
Poverty-income ratio	1.033	0.917–1.164	0.566

## Discussion

The present study evaluated the association between systemic inflammatory indices and breast cancer using a large nationally representative NHANES cohort. Exploratory unweighted comparisons demonstrated higher median inflammatory index values among participants with breast cancer; however, these differences were not independently associated with breast cancer in survey-weighted multivariable analyses. Increasing age consistently remained the strongest independent factor associated with breast cancer across all regression models.

Several NHANES-based studies have explored the relationship between systemic inflammatory indices and breast cancer. These studies consistently suggested a potential association between elevated markers such as SII, NLR, and PLR and breast cancer risk. Large datasets from NHANES cycles spanning 1999-2018 and 2001-2018 have demonstrated that higher inflammatory indices are associated with increased breast cancer prevalence. SII often emerged as a particularly strong predictor in older populations and across higher-risk strata [22-25]. However, findings across studies are not fully consistent. While earlier NHANES analyses reported independent associations between elevated inflammatory indices and breast cancer even after multivariable adjustment. Some studies described nonlinear relationships (including inverse L-shaped associations) and differential predictive performance of PLR and SII [23,24]. In contrast, other studies have identified SII as an independent predictor of breast cancer prevalence with significant discriminatory ability on ROC analysis [25]. This discrepancy from several previous NHANES reports may reflect differences in study periods, population characteristics, outcome definitions, and particularly variations in statistical modeling strategies. The attenuation of associations observed after multivariable adjustment suggests that the elevated inflammatory indices identified in unadjusted analyses may have been largely explained by differences in age and other demographic, socioeconomic, and lifestyle-related factors between participants with and without breast cancer. Given the well-established relationship between aging, chronic low-grade inflammation, and cancer risk, adjustment for these covariates may have accounted for a substantial proportion of the observed crude associations. It is also possible that residual confounding from unmeasured breast cancer risk factors contributed to the findings. An additional consideration is that the present study evaluated self-reported prevalent breast cancer rather than incident disease. Consequently, inflammatory marker levels measured at survey participation may not reflect inflammatory status at the time of cancer development and may instead be influenced by survivorship-related factors, prior treatment, or lifestyle modifications following diagnosis. This methodological difference may partly explain discrepancies between our findings and studies evaluating incident breast cancer risk.

Hospital-based studies have also highlighted the prognostic relevance of systemic inflammatory indices in breast cancer. Ciurescu et al. reported that elevated preoperative SII levels were associated with advanced tumor stage and adverse prognostic characteristics, while multivariable analysis suggested additional predictive value [26]. These findings may indicate that inflammatory indices have greater utility in assessing tumor burden, disease aggressiveness, or prognosis rather than population-level breast cancer prevalence. This interpretation is further supported by a meta-analysis involving breast cancer patients receiving endocrine therapy, where lower NLR and PLR values were associated with improved progression-free and overall survival outcomes [27]. Unlike prognostic studies conducted among patients with established malignancy, the present study evaluated breast cancer prevalence within a general population cohort, which may partly explain the absence of independent associations after adjustment. Furthermore, because NHANES captures participants with a prior history of breast cancer rather than newly diagnosed cases, survivorship bias may have influenced the observed associations. Participants who survived long enough to participate in NHANES may differ from the broader breast cancer population with respect to inflammatory status, treatment exposure, and overall health characteristics.

Another retrospective study evaluating inflammatory indices in breast cancer patients demonstrated that elevated NLR was significantly associated with higher tumor grade and HER2 expression status, suggesting a relationship between systemic inflammation and tumor aggressiveness [28]. The authors also reported moderate discriminatory performance of NLR for predicting tumor grade. Such observations further support the possibility that inflammatory indices may be more closely linked with disease severity, progression, and tumor biology rather than functioning as independent indicators of breast cancer prevalence within the general population. Furthermore, although the overall cohort was large and nationally representative, the relatively limited number of breast cancer cases may have reduced statistical power to detect modest independent associations between inflammatory indices and breast cancer status after multivariable adjustment. Residual confounding also cannot be excluded because several established breast cancer risk factors, including BRCA1 / BRCA2 mutation, hormone therapy use, family history of breast cancer, and genetic susceptibility, were not available for incorporation into the present analysis. Furthermore, the outcome reflected a self-reported history of breast cancer rather than incident disease. Consequently, survivorship-related effects, prior treatment exposure, and post-diagnosis lifestyle modifications may have influenced inflammatory marker levels measured at the time of survey participation.

Limitations

Several limitations should be considered while interpreting the findings of the present study. First, the cross-sectional design of NHANES limits the ability to establish temporal or causal relationships between inflammatory indices and breast cancer status. Second, breast cancer history in NHANES was based on self-reported physician diagnosis, which may introduce recall bias or misclassification. Third, information regarding tumor subtype, disease stage, treatment status, duration since diagnosis, recurrence, and survival outcomes was not available, limiting detailed oncological interpretation of inflammatory markers. Fourth, inflammatory indices were calculated from single-time-point laboratory measurements and therefore may not accurately reflect long-term inflammatory status. Fifth, residual confounding cannot be excluded because several established breast cancer risk factors, including BRCA1/BRCA2 mutation status, hormone replacement therapy, family history of breast cancer, and genetic susceptibility, were not available for inclusion in the analysis. Sixth, the use of complete-case analysis may have introduced selection bias if excluded participants differed systematically from those included. Seventh, the outcome reflected a self-reported history of breast cancer rather than incident disease; therefore, inflammatory marker levels measured at the time of survey participation may not represent inflammatory status at the time of cancer development. Furthermore, reverse causation and survivorship bias are possible because NHANES captures participants with a prior history of breast cancer who survived long enough to participate in the survey. Prior cancer treatment, survivorship-related factors, and lifestyle modifications following diagnosis may have influenced inflammatory marker levels. Sensitivity analyses were not performed; therefore, the robustness of findings under alternative modeling approaches could not be evaluated. Finally, although NHANES provides nationally representative data, the relatively small number of participants with breast cancer (n=150) may have limited statistical power to detect modest independent associations between inflammatory indices and breast cancer status after multivariable adjustment.

Recommendations

Further large-scale prospective longitudinal studies should be conducted to evaluate the temporal relationship between systemic inflammatory indices and breast cancer development. Future research should incorporate detailed oncological characteristics, including tumor subtype, disease stage, treatment status, and survival outcomes, to better clarify the diagnostic and prognostic significance of NLR, PLR, and SII in breast cancer. Studies using repeated inflammatory marker measurements over time may also provide better insight into the role of chronic systemic inflammation in breast cancer biology. Additionally, standardized statistical approaches incorporating survey-weighted analyses are recommended in future NHANES-based studies to improve methodological consistency and comparability across studies.

## Conclusions

In this large population-based cross-sectional analysis of NHANES 2021-2023 data, higher unweighted median inflammatory index values were observed among participants with breast cancer; however, no independent associations were identified after survey-weighted multivariable adjustment. Increasing age consistently emerged as the strongest independent factor associated with breast cancer across all adjusted regression models. Further prospective longitudinal studies are needed to clarify the diagnostic, prognostic, and predictive value of inflammatory indices in breast cancer.
